# A Lower Olfactory Capacity Is Related to Higher Circulating Concentrations of Endocannabinoid 2-Arachidonoylglycerol and Higher Body Mass Index in Women

**DOI:** 10.1371/journal.pone.0148734

**Published:** 2016-02-05

**Authors:** Antoni Pastor, Fernando Fernández-Aranda, Montserrat Fitó, Susana Jiménez-Murcia, Cristina Botella, Jose M. Fernández-Real, Gema Frühbeck, Francisco J. Tinahones, Ana B. Fagundo, Joan Rodriguez, Zaida Agüera, Klaus Langohr, Felipe F. Casanueva, Rafael de la Torre

**Affiliations:** 1 Integrative Pharmacology and Systems Neuroscience Research Group, Neuroscience Research Program, IMIM (Hospital de Mar Medical Research Institute), Barcelona, Spain; 2 Department of Psychiatry, University Hospital of Bellvitge-IDIBELL, Barcelona, Spain; 3 Cardiovascular Risk and Nutrition Research Group, Inflammatory and Cardiovascular Disorders Research Program, IMIM (Hospital del Mar Medical Research Institute), Barcelona, Spain; 4 Department of Clinical Sciences, School of Medicine, University of Barcelona, Barcelona, Spain; 5 Department of Basic Psychology, Clinic and Psychobiology, University Jaume I, Castelló, Spain; 6 Department of Diabetes, Endocrinology and Nutrition, Institut d’Investigació Biomèdica de Girona (IdlBGi), Hospital Dr Josep Trueta, Girona, Spain; 7 Department of Endocrinology and Nutrition, Clínica Universidad de Navarra, University of Navarra, IdiSNA, Pamplona, Spain; 8 Department of Diabetes, Endocrinology and Nutrition, Hospital Clínico Universitario Virgen de Victoria, Málaga, Spain; 9 Department of Statistics and Operations Research, Universitat Politècnica de Catalunya, Barcelona, Spain; 10 Endocrine Division, Complejo Hospitalario U. de Santiago, Santiago de Compostela University, Santiago de Compostela, Spain; 11 Department of Pharmacology, School of Medicine, Universitat Autònoma de Barcelona, Barcelona, Spain; 12 CIBER Fisiopatología Obesidad y Nutrición (CIBERObn), Instituto Salud Carlos III, Madrid, Spain; 13 Department of Experimental and Health Sciences, Universitat Pompeu Fabra, Barcelona, Spain; INSERM, FRANCE

## Abstract

The endocannabinoid (eCB) system can promote food intake by increasing odor detection in mice. The eCB system is over-active in human obesity. Our aim is to measure circulating eCB concentrations and olfactory capacity in a human sample that includes people with obesity and explore the possible interaction between olfaction, obesity and the eCB system. The study sample was made up of 161 females with five groups of body mass index sub-categories ranging from under-weight to morbidly obese. We assessed olfactory capacity with the “Sniffin´Sticks” test, which measures olfactory threshold-discrimination-identification (TDI) capacity. We measured plasma concentrations of the eCBs 2-arachidonoylglycerol (2-AG) and N-arachidonoylethanolamine or anandamide (AEA), and several eCB-related compounds, 2-acylglycerols and N-acylethanolamines. 2-AG and other 2-acylglycerols fasting plasma circulating plasma concentrations were higher in obese and morbidly obese subjects. AEA and other N-acylethanolamine circulating concentrations were lower in under-weight subjects. Olfactory TDI scores were lower in obese and morbidly obese subjects. Lower TDI scores were independently associated with higher 2-AG fasting plasma circulating concentrations, higher %body fat, and higher body mass index, after controlling for age, smoking, menstruation, and use of contraceptives. Our results show that obese subjects have a lower olfactory capacity than non-obese ones and that elevated fasting plasma circulating 2-AG concentrations in obesity are linked to a lower olfactory capacity. In agreement with previous studies we show that eCBs AEA and 2-AG, and their respective congeners have a distinct profile in relation to body mass index. The present report is the first study in humans in which olfactory capacity and circulating eCB concentrations have been measured in the same subjects.

## Introduction

Food intake and body weight regulation are processes highly controlled by brain neural systems that integrate a multitude of signals and hormones relaying information about both the internal state and the environment [1]. The high prevalence of obesity in modern industrialized societies [2] has been linked to changes in environmental factors together with a sedentary lifestyle. A widespread availability of energy-dense and highly palatable foods and the increased presence of powerful food cues override homeostatic satiety signals by influencing cortico-limbic brain areas concerned with learning, memory, reward, and emotion [3]. One of such areas is the olfactory system. The sense of smell is essential for human flavor perception [4] and it contributes to our food choices and eating behavior by participating in the hedonic evaluation of foods [5,6]. Olfaction influences both appetite and satiety in humans [7]. A high reactivity to olfactory food cues has been observed in over-weight children [8]. Food-related odors activate brain reward circuits during hunger, and the more active areas are different in obese and normal-weight subjects [9]. Converging evidence also suggests that the olfactory system is intimately linked to neural systems that regulate food intake by acting as a sensor of the metabolic state [10]. This is supported by the presence in the olfactory bulb of receptors of several hormones and neuromodulators that regulate food intake, among them: insulin, leptin, ghrelin, orexin or endocannabinoids [10]. It has been recently demonstrated that the endocannabinoid (eCB) system, a key homeostatic and hedonic modulator of food intake [11], promotes eating in fasted mice by enhancing the sense of smell [12]. This important finding raises issues regarding the role of odor perception in eating behavior and obesity. As reviewed by Kirkham [13], exogenous and endogenous cannabinoids stimulate food intake by increasing the motivation to eat and by amplifying the palatability, or orosensory reward of food. Administration of the cannabinoid delta-9-tetrahidrocannabinol (THC) improves taste and smell and the enjoyment of food in cancer patients [14]. In obesity, the central and peripheral eCB system becomes over-active [15,16]. Peripheral signals that regulate food intake like insulin, leptin or ghrelin that interact with the eCB system and which are also altered in obesity [17] seem to modulate olfactory sensitivity[10,18–20].

Olfactory capacity decreases with the increase in body mass index [21]. The reason is not well understood but it may be related to cognitive deficits associated to obesity [22], since olfactory capacity can predict cognitive decline [23]. Olfaction is processed in the limbic area of the brain where emotion and memory are stored. Differently to other senses that need the thalamus relay, the olfactory sense is unique since it is the only sense that has a direct connection to the higher cognitive centers of the brain. The olfactory bulb has direct connections to the piriform cortex. The piriform cortex and the amygdala both project to the orbitofrontal cortex (OFC) which with the amygdala is involved in emotion and associative learning, and to the entorhinal/hippocampal system which is involved in long-term memory including episodic memory [24–27]. Olfaction has two components, detection and perception. First, the odorants are detected on the nasal cavity by specific receptor cells. Second, the information is sent and processed in the brain. Perception is much more complex than detection because it involves memory and emotion in the identification of an odor [4,26].

Our aim is to measure circulating eCB concentrations and olfactory capacity in a human sample that includes people with obesity and explore the possible interaction between olfaction, obesity and the eCB system. In the present report we show that obese subjects have a reduced olfactory capacity and that higher eCB fasting plasma circulating concentrations in obesity are associated to a reduced olfactory capacity.

## Methods

### Subjects

The study sample was made up of 161 females (aged 18–65 years) with body mass indices (BMI) ranging from under-weight to morbid obesity. The characteristics of the study subjects are presented in Table 1. Seven centers, all involved in the CIBERObn (Spanish Biomedical Research Centre in Physiopathology of Obesity and Nutrition, www.ciberobn.com), participated. Only women were selected for this study. The population of subjects from the present study comes from a larger clinical cohort for the study of eating disorders in women. Obese participants (BMI>30 Kg/m^2^) were patients who had been consecutively referred to the clinics mentioned above. Recruitment of the non-obese participants (BMI<30 Kg/m^2^) took place by means of word-of-mouth and advertisements at local universities. Non-obese participants were from the same catchment area as the clinical groups. The selection was based on BMI. Exclusion criteria included: a) being male, b) a history of chronic medical illness, c) the use of psychoactive medication and drugs, d) Age under 18 or over 65 years. Subjects diagnosed with eating disorders, such as anorexia, bulimia, or binge eating disorder, were excluded from this sample. Enrolment in the study was between January 2010 and September 2012. All participants gave written and signed informed consent, the study was conducted according to the Declaration of Helsinki and the Ethics Committee of all the institutions involved [Comissió Deontológica de la Universitat Jaume I, Subcomisión Clínica del Hospital Universitario “Virgen de la Victoria”, Málaga, Comite Etic de Investigacio Clinica Hospital Universitari de Girona Doctor Josep Trueta (048/10), Comite Etico de Investigacion Clinica del Consorci Mar Parc de Salut de Barcelona-Parc de Salut Mar (2010/3914/I), Comité de Etica de la Investigación Universidad de Navarra (110/2010) and Comité Etico de Investigación Clínica del Hospital Universitari de Bellvitge (307/06)] approved the study.

**Table 1:** Characteristics of the study subjects.

	UW (n = 18)	NW (n = 70)	OW (n = 13)	OB (n = 26)	MO (n = 34)
	BMI<19 kg/m^2^	19≤BMI<25 kg/m^2^	25≤BMI<30 kg/m^2^	30≤BMI<40 kg/m^2^	BMI≥40 kg/m^2^
**Age (yrs)**	24.0 (7.45)	27.4 (7.36)	33.4 (7.64)	47.3 (11.1)	43.5 (11.2)
**Height (cm)**	164 (7.99)	166 (6.40)	162 (4.11)	161 (6.27)	161 (7.36)
**Weight (Kg)**	48.9 (5.20)	59.9 (6.31)	70.9 (4.67)	92.0 (8.44)	122 (17.8)
**BMI (Kg/m**^**2**^**)**	18.1 (0.64)	21.7 (1.62)	26.9 (0.94)	35.5 (2.53)	46.7 (5.10
**% fat mass**	18.7 (4.06)	27.0 (4.48)	33.6 (3.59)	42.1 (6.14)	46.4 (4.19)
**LDL-C (mg/dL)**	84.5 (28.9)	92.7 (23.8)	106 (26.1)	116 (33.5)	107 (24.7)
**HDL-C (mg/dL)**	55.4 (16.0)	58.1 (10.3)	58.5 (16.0)	47.6 (7.39)	43.8 (8.98)
**Total-C (mg/dL)**	154 (43.1)	166 (31.0)	180 (36.8)	187 (37.8)	179 (33.7)
**Triglycerides (mg/dL)**	65.9 (25.9)	77.4 (29.4)	84.9 (44.6)	138 (85.5)	148 (63.3)
**Glucose (mg/dL)**	86.6 (9.34)	86.0 (19.8)	87.6 (4.14)	102 (25.5)	101 (18.4)

Data are mean (standard deviation), under-weight (UW), normal-weight (NW), over-weight (OW), obese (OB), morbidly obese (MO), body mass index (BMI), low density lipoprotein cholesterol (LDL-C), high density lipoprotein cholesterol (HDL-C), total cholesterol (Total-C)

### Anthropometry

For the purpose of the study, the following measures were considered: height (m), weight (kg), BMI (kg/m^2^), and %body fat. Body composition was assessed using a Tanita Multi-Frequency Body Composition Analyzer MC-180MA (Tanita Corp., Tokyo, Japan). Tanita uses the non-invasive technique of bioelectrical impedance analysis (BIA) that predicts from a series of equations total body water content and free fat mass, from which fat mass can be calculated.

### Lipid profile

EDTAK2 plasma glucose, total cholesterol (Total-C), and triglycerides were determined by enzymatic methods, high-density lipoprotein cholesterol (HDL-C) by an accelerator selective detergent method in a PENTRA-400 autoanalyzer (ABX-HORIBA Diagnostics, Montpellier, France). Low-density lipoprotein cholesterol (LDL-C) was calculated by the Friedewald equation whenever triglycerides were <300 mg/dL.

### Quantification of eCBs and related compounds

The eCBs anandamide (AEA) and 2-arachidonoylglycerol (2-AG) were quantified in plasma of participants. In addition, the following eCB chemically related compounds, N-acylethanolamines and 2-acylglycerols, were quantified in plasma: palmitoylethanolamide (PEA), linoleoylethanolamide (LEA), oleoylethanolamide (OEA), docosahexaenoylethanolamide (DHEA), 2-linoleoylglycerol (2-LG), and 2-oleoylglycerol (2-OG). Blood samples were collected from subjects between 8 and 9 am after at least 12 hours of fasting, and on the same day that the olfactory tests were undertaken. Blood was collected in 10 mL K2E 18.0 mg (EDTA) BD Vacutainer tubes. Blood tubes were centrifuged at 1700 g at 4°C for 15 min. Plasma was separated, distributed in aliquots, and stored at -80°C until analysis. The extraction and analysis of plasma eCBs and eCB-related compounds was performed by a validated method previously described [28]. Briefly, aliquots of 0.5 mL of plasma of the study subjects were transferred to 12 mL glass tubes, spiked with isotope-labeled internal standards, diluted with 0.1M ammonium acetate buffer (pH 4.0), and extracted with *tert*-butyl methyl ether. The organic layers were separated into clean 12mL glass tubes and placed in a water bath (29°C) under a stream of nitrogen. The dry organic extracts were reconstituted in 100 μL of a mixture water: acetonitrile (10:90, v/v) with 0.1% formic acid (v/v) and transferred to HPLC vials. Twenty μL were injected into the LC/MS-MS system. An Agilent 6410 triple quadrupole (Agilent Technologies, Wilmington, DE) coupled to a chromatographic system consisting in a 1200 series binary pump, a column oven and a cooled autosampler (4°C) were used. Chromatographic separation was carried out by gradient chromatography with a Waters C18-CSH column (3.1 x 100 mm, 1.8 μm particle size) maintained at 40°C with a mobile phase flow rate of 0.4 mL/min. The composition of the mobile phase was: A: 0.1% (v/v) formic acid in water; B: 0.1% (v/v) formic acid in acetonitrile. The electrospray ion source was set on the positive ionization mode. The mass spectrometry detection was done by single reaction monitoring (SRM). Quantification was done by isotope dilution. Isotope-labeled internal standards were obtained from Cayman Chemical (Ann Arbor, MI) and solvents were from Merck (Darmstadt, Germany). 2-acylglycerols were reported as the sum of isomer 1 and isomer 2 due to the instability of isomer 2 to isomerization. Quantification was done by isotope dilution.

### Olfactory Capacity tests

Olfactory capacity was assessed by the “Sniffin´Sticks” test (Burghart Messtechnik GmBh, Wedel, Germany), which evaluates chemosensory performance based on pen-like odor-dispensing devices. The test has been previously described and validated [29] and it is deemed suitable for the routine clinical assessment of olfactory performance. It is made up of three sub-tests: odor threshold (OT), odor discrimination (OD), and odor identification (OI). OT is the lowest concentration of a certain odor compound that is perceivable by the human sense of smell. OD and OI show the capacity to differentiate and identify odorants, respectively. OD and OI are more related to cognitive aspects of olfaction, while OT is more sensorial [30]. OT was assessed with *n*-butanol, while OD and OI were assessed with sixteen common fragrances, such as peppermint, orange, leather, cinnamon, banana, garlic, lemon, rose, coffee, apple, clove, pineapple, aniseed and fish [29]. Subjects were assessed individually in a well-ventilated room and they wore eye masks to perform the test. Subjects were requested not to smoke, chew gum or having eaten any products during the previous one hour at the start of the test. The tests were performed in the time period after breakfast and before lunch and on the same day that blood was collected for eCB analysis. A trained researcher carried out the tests in the following order: OT, OD and OI.

#### Olfactory threshold (OT) test

Using a triple-forced choice paradigm, detection thresholds were measured by employing a single staircase method. Three pens were presented in a randomized order, two pens contained odorless samples, and the third contained an odorant sample at a particular dilution. A total of 16 odor concentrations were tested. The task of the subject was to indicate which pen contained the odorant. Concentration of the odorant was augmented if the subject chose an odorless pen and reduced if the correct pen was recognized twice, which triggered a reversal of the staircase. The mean of the last four staircase reversal points of a total of seven reversals was used as the threshold estimate. The range of this score is from 0 to 16 points. The higher is the score the higher is olfactory threshold capacity.

#### Olfactory discrimination (OD) test

The subjects were asked to discriminate between 16 triplets of odors. In each group two odors were identical and one odor was different and the task was to recognize the pen in which the odor was different. The total score was the sum of correct responses, which can range from 0 to 16 points. The higher is the score the higher is olfactory discrimination capacity.

#### Olfactory identification (OI) test

A pen with an odor was presented to the subject. Subjects were asked to identify the odor, by choosing it in a card from a list of four descriptors, only one of which correctly identified the odor. For this purpose the eye mask was removed (only for reading the card). The total score was the sum of correct responses, which can range from 0 to 16 points. The higher is the score the higher is olfactory identification capacity.

#### Threshold-discrimination-identification (TDI) score

The sum of the scores from the three subtests results in the composite olfactory threshold-discrimination-identification (TDI) score, which can range from 0 to 48 points. The higher is the score the higher is olfactory capacity. TDI declines with age, the strongest decrease is observed in individuals >55 years [31]. Normosmia, or normal olfactory function is defined for TDI scores higher than 30.3 points, hyposmia, or decrease in olfactory capacity is defined for TDI scores lower than 30.3, and anosmia, or functional loss of olfactory capacity is defined for TDI scores lower than 16.5.

### Statistical analysis

Characteristics of the five BMI sample groups are presented by the mean and the standard deviation. Boxplots (Tukey) were generated with software GraphPad Prism 5. Statistical analysis was performed with the software packages SPSS (version 18) and R (version 3.2.1). ANCOVA models were employed to compare the BMI groups with respect to eCB concentrations and olfactory scores adjusting for age. In the framework of this model the Tukey test was used for the post-hoc pairwise comparisons. The correlations between the olfactory variables, on one hand, and the other study variables, on the other hand, were estimated using Pearson's correlation coefficient. In addition, the association of TDI scores with BMI, %body fat and 2-AG was studied by means of ANCOVA models that controlled for age, smoking (yes/no), menstruation (normal/irregular/menopause), and the use of contraceptives (yes/no) as all these variables could affect olfactory capacity. Both the estimated parameter (B) of each variable of interest and the coefficient of determination (R^2^), which represents the percentage of variability of TDI explained by the models, are presented.

## Results

### Differences in eCB concentrations between BMI groups

**Fig 1:**
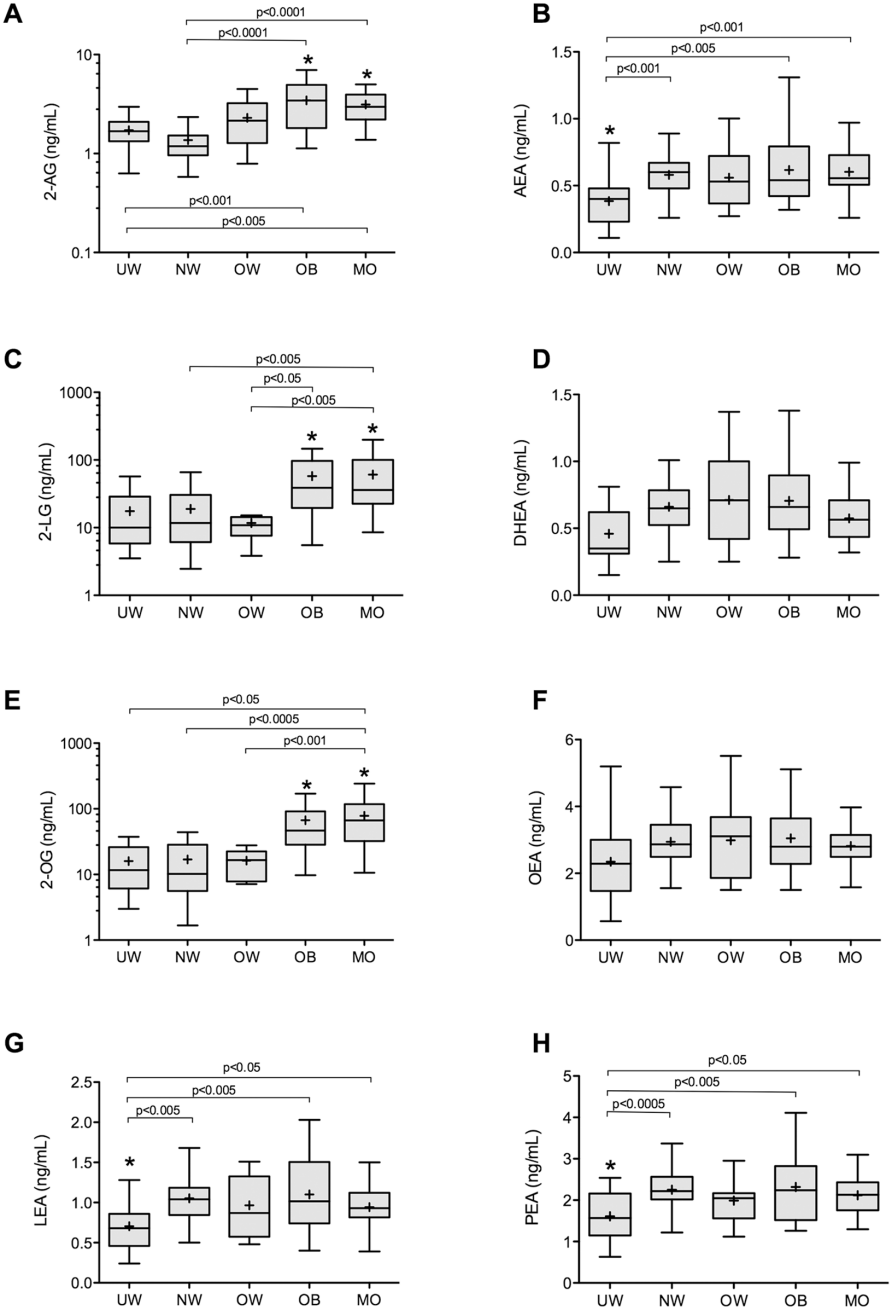
Endocannabinoid and related compounds plasma concentrations. Box plots of plasma concentrations of 2-arachidonoyl glycerol (A), anandamide (B), 2-linoleoylglycerol (C), docosahexaenoylethanolamide (D), 2-oleoylglycerol (E), oleoylethanolamide (F), linoleoylethanolamide (G), and palmitoylethanolamide (H) of subjects from each of the body mass index (BMI) sub-groups. The median is represented as a line within the box plot and the mean is represented as a + sign. Significant differences (p<0.05) of endocannabinoids and related compounds concentrations between BMI subgroups are represented by asterisks (*).

The concentrations of eCBs and related compounds of each BMI sample group are presented in Fig 1. Plasma concentrations of 2-monoacylglycerols 2-AG, 2-LG and 2-OG were significantly elevated in obese and morbidly obese subjects, while plasma concentrations of N-acylethanolamines AEA, LEA and PEA were significantly lower in under-weight subjects.

### Olfactory capacity, BMI and eCBs

**Fig 2:**
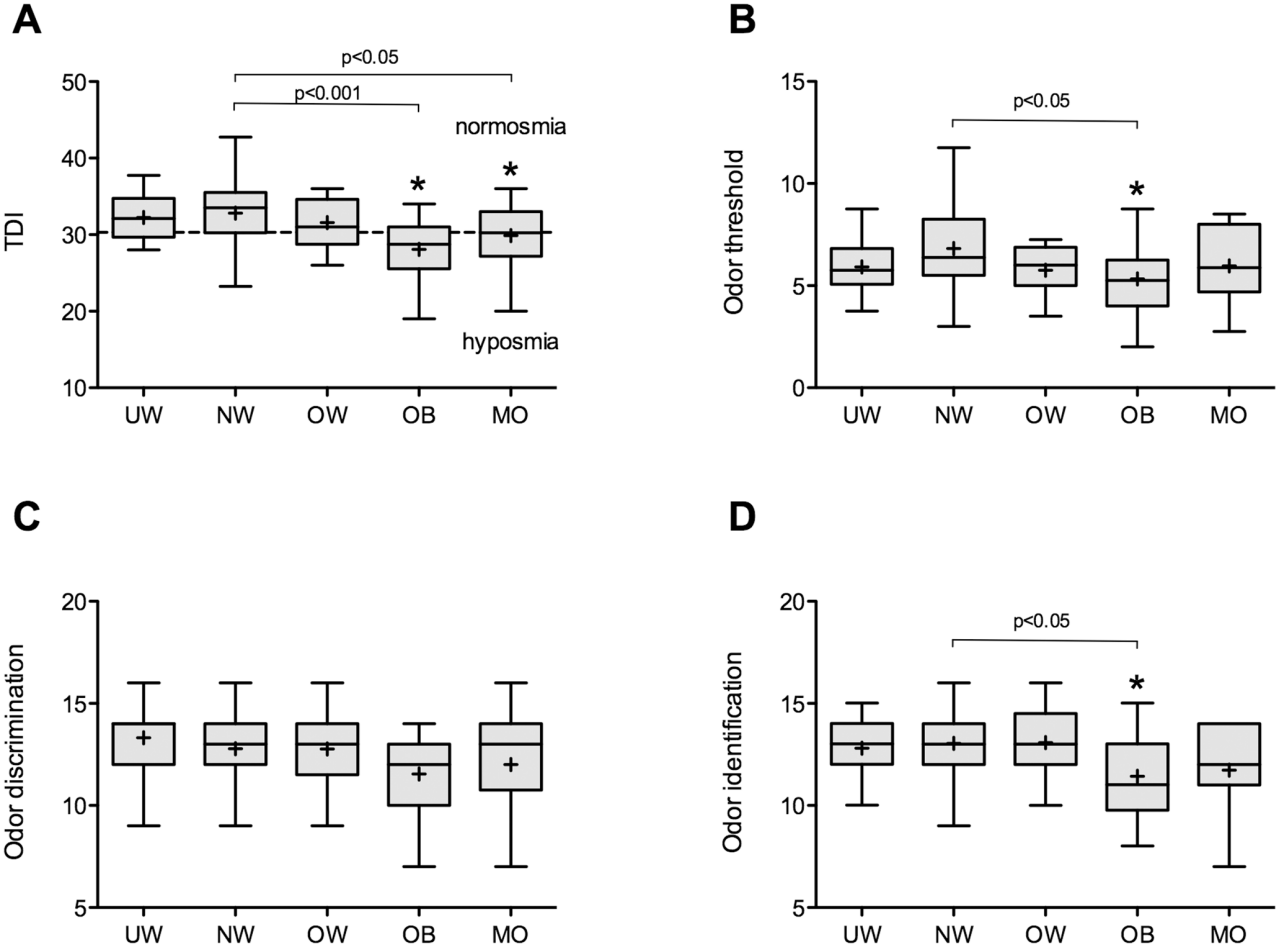
Olfactory scores. Box plots of odor threshold-discrimination-identification (TDI) scores (A), odor threshold scores (B), odor discrimination scores (C), and odor identification scores (D) of subjects from each of the body mass index (BMI) sub-groups. The median is represented as a line within the box plot and the mean is represented as a + sign. Discontinuous line in (A) represents the TDI score of 30.3 that separates normosmia from hyposmia. Significant differences (p<0.05) of olfactory scores between BMI sub-groups are represented asterisks (*).

Olfactory scores of each BMI sample group are presented in Fig 2. TDI scores are shown in relation to the TDI score of <30.3, typically used in clinics for hyposmia diagnostic [31]. TDI scores were significantly lower in obese and morbidly obese subjects. With respect to the olfactory sub-tests OT and OI scores were significantly lower in obese subjects but not in morbidly obese subjects.

Next, we assessed the correlation of olfactory scores with the other study variables. TDI scores negatively correlated with age, BMI, %body fat, and fasting plasma concentrations of 2-AG, 2-LG, 2-OG, triglycerides and glucose (Table 2). We note that triglycerides and glucose did not correlate with OT.

**Table 2:** Olfactory scores correlations.

	Odor threshold	Odor discrimination	Odor identification	TDI
**Age (yrs)**	r = -0.16, p = 0.049	r = -0.21, p = 0.008	r = -0.25, p = 0.002	r = -0.29, p<0.001
**BMI (Kg/m**^**2**^**)**	r = -0.17, p = 0.030	r = -0.20, p = 0.011	r = -0.31, p<0.001	r = -0.32, p<0.001
**%body fat**	r = -0.21, p = 0.009	r = -0.23, p = 0.004	r = -0.27, p = 0.001	r = -0.33, p<0.001
**2-AG (ng/mL)**	r = -0.23, p = 0.004	r = -0.23, p = 0.004	r = -0.23, p = 0.003	r = -0.33, p<0.001
**2-LG (ng/mL)**	r = -0.15, p = 0.063	r = -0.20, p = 0.015	r = -0.20, p = 0.017	r = -0.26, p = 0.001
**2-OG (ng/mL)**	r = -0.14, p = 0.080	r = -0.18, p = 0.025	r = -0.18, p = 0.024	r = -0.24, p = 0.003
**Triglycerides (mg/dL)**	r = -0.10, p = 0.209	r = -0.15, p = 0.065	r = -0.18, p = 0.025	r = -0.21, p = 0.010
**Glucose (mg/dL)**	r = -0.08, p = 0.307	r = -0.19; p = 0.023	r = -0.21; p = 0.009	r = -0.23; p = 0.005

Odor threshold-discrimination-identification score (TDI), body mass index (BMI), 2-arachidonoylglycerol (2-AG), 2-linoleoylglycerol (2-LG), 2-oleoylglycerol (2-OG)

In addition to age olfaction may be affected by other factors such as smoking. status. Estrogen levels may also affect olfaction. Thus, menopause, normal or irregular menstruation and the use of contraceptives are possible cofounding factors. The mean olfactory scores of subjects categorized with these cofounding factors are shown in Table 3.

**Table 3:** Olfactory scores of study subjects in relation to different cofounding factors.

	TDI	Odor threshold	Odor discrimination	Odor identification
**Normal menstruation**	32.1 (4.02)	6.62 (2.22)	12.7 (1.84)	12.8 (1.82)
**Irregular menstruation**	30.7 (4.86)	6.08 (1.90)	12.4 (2.80)	12.2 (2.09)
**Menopause**	28.3 (3.93)	5.47 (1.51)	11.8 (2.25)	11.1 (2.55)
**Do not use oral contraceptives**	31.2 (4.63)	6.38 (2.27)	12.4 (2.18)	12.4 (2.14)
**Use of oral contraceptives**	31.5 (3.29)	6.21 (1.56)	12.7 (1.61)	12.6 (1.75)
**Non-Smokers**	31.3 (4.29)	6.24 (1.70)	12.4 (2.14)	12.6 (1.90)
**Smokers**	31.9 (4.18)	6.33 (2.62)	12.9 (1.70)	12.6 (2.05)

Data are mean (standard deviation); Odor threshold-discrimination-identification (TDI) score

We studied the association of TDI with the other study variables by means of ANCOVA models that controlled for age, smoking, menstruation, and the use of contraceptives. We found that lower TDI scores were independently associated with higher BMI [B = -0.09 (-0.17,-0.01; 95% CI), p = 0.026, R^2^_model_ = 0.16], higher %body fat [B = -9.97 (-18.0,-1.92; 95% CI), p = 0.016, R^2^_model_ = 0.17], and higher 2-AG fasting plasma circulating concentrations [B = -0.83 (-1.45,-0.20; 95% CI), p = 0.010, R^2^_model_ = 0.17]. In this sample olfactory scores negatively correlated with age and BMI (Table 2). Although the statistical adjustment for age is valid we also evaluated the olfactory scores and 2-AG concentrations in a sub-sample of individuals that were selected to have homogeneity in the age of BMI groups. A subsample of 54 individuals was selected from the study sample with ages comprised between 30–45 years old (Table 4).

**Table 4:** OIfactory scores and endocannabinoid concentrations in a subsample of individuals aged between 30–45 years old.

	Non-obese (n = 30)	Obese (n = 24)	p
	BMI < 30 kg/m^2^	BMI ≥ 30 Kg/m^2^	
**Age (yrs)**	35.0 (3.97)	36.5 (4.56)	0.211
**BMI (Kg/m**^**2**^**)**	22.6 (7.99)	41.7 (6.50)	<0.001
**% body fat**	27.4 (5.51)	44.6 (5.47)	<0.001
**2-AG (ng/mL)**	1.41 (0.79)	3.05 (1.41)	<0.001
**TDI**	33.7 (4.67)	29.6 (3.81)	0.001
**Odor threshold**	7.31 (2.85)	5.92 (1.70)	0.039
**Odor discrimination**	13.3 (1.82)	11.5 (2.19)	0.002
**Odor identification**	13.2 (1.79)	12.2 (1.74)	0.053

Data are mean (standard deviation); Odor threshold-discrimination-identification (TDI) score; Unpaired t-test (p)

Results on Table 4 confirm that in two different BMI groups homogeneous by age, obese subjects (BMI ≥ 30 Kg/m^2^) have a lower olfactory capacity and higher 2-AG fasting plasma circulating concentrations than non-obese subjects. The BMI sub-categories within these groups were not considered for this analysis to not lose statistical power.

## Discussion

Results of the present report show that obese subjects have a lower olfactory capacity than non-obese ones and that higher 2-AG fasting plasma circulating concentrations are also linked to a lower olfactory capacity. In agreement with previous studies we show that eCBs AEA and 2-AG, and their respective congeners have a distinct profile in relation to body mass index. The study sample comprised women with a wide range of BMI from under-weight to morbidly obese. Age is the most important factor that affects olfaction. We note that the mean age of the groups was lower for lower BMI categories and higher for higher BMI categories. These differences were taken into account in the statistical analysis. In the comparison between eCB concentrations and olfactory scores between BMI groups the statistical analysis were adjusted for age. In the ANCOVA models the associations between BMI, %body fat, 2-AG concentrations and TDI were adjusted by age and other possible cofounding factors that included smoking status, normal or irregular menstruation, menopause and use of oral contraceptives. Our findings were confirmed in a sub-sample of individuals aged 30–45 years old in which the age of two different BMI groups, obese and non-obese was homogeneous.

Olfaction seems to be affected by the nutritional status [10]. We performed the olfactory tests in a relatively low hunger state, which is the time period between breakfast and lunch and subjects could not have eaten at the previous hour of the start of the test. A high hunger state increases olfactory sensitivity [32,33] and food palatability [32]. It is noteworthy that studies have reported differences in olfactory threshold under hunger or satiety between neutral and food odors [33] [34] or in the pleasantness of odors [34]. Interestingly, in a recent study it was reported that obese subjects have a greater sensitivity and preference for the odor of chocolate than non-obese subjects [35]. This is in contrast to our results in which we used the neutral odorant *n*-butanol for the OT test. These differences are possibly due to the different hedonic value of odors, especially the food-related ones. For instance, it also has been reported that obese subjects perceive the odor of black pepper oil as less pleasant than non-obese subjects [36].

Obese and morbidly subjects had a lower olfactory capacity than normal-weight subjects. OI and OT were the olfactory domains more affected by an increase of BMI. In this study we did not observe a worse olfactory capacity in morbid obese vs obese subjects as reported previously [21]. The decrease in olfactory capacity in obesity may be explained by a loss of olfactory neurons due to a high fat diet, as it has been observed in mice [37]. It is noteworthy that in mice models of diet-induced obesity, the levels of eCBs, the expression of eCB biosynthetic and inactivating enzymes and the expression of eCB receptors are altered in the hippocampus [38] [39], a region that participates in the formation of odor memories, mediates hedonic aspects of eating, and influences cognitive processes. It has been hypothesized that a Western diet rich in saturated fat and sugar leads to obesity due to neurobiological changes in the hippocampus that affect cognitive function, specifically in memory and learning functions necessary for the inhibitory control in response to food cues [40]. Cognitive functions related to olfaction are also relevant in humans with regard to learning a flavor during ingestion [6]. Finally, it has been reported previously that 2-AG circulating levels inversely correlate with cognitive flexibility [41].

The over-activation of the eCB system in obesity may lead to changes in olfactory perception and eating behavior. A previous report has shown that cannabinoids decrease odor threshold and stimulate food intake in fasted mice by means of olfactory processes [12]. In the present study, we found that a reduced olfactory capacity is related to high 2-AG fasting plasma circulating concentrations and high BMI. Permanent high eCB concentrations associated to obesity may lead to adaptive responses such as resistance to eCB signaling. For instance, a chronic exposure to a high-fat, palatable diet in mice has been shown to decrease the expression of the CB1 receptor in the nucleus accumbens shell, the hedonic “hotspot”, and in the hippocampus [42]. An alteration of reward pathways has been postulated as one possible cause for obesity [43]. In this work we did not quantify food enjoyment, but a loss of flavor due to a reduced olfactory capacity could shift the diet to eating more high caloric and palatable foods [44]. In addition, satiety mechanisms that rely on the olfactory system, such as olfactory sensory-specific satiety may be affected [45].

2-AG circulating concentrations, but not AEA ones, were inversely associated with olfactory capacity. 2-AG was also the eCB associated with obesity. Our results are in agreement with previous studies that relate 2-AG but not AEA with %visceral fat and dyslipidemia [46–48]. Notably, olfactory capacity was also inversely associated to %body fat. Although 2-AG and AEA are both agonists of CB1 receptors and they share the backbone of arachidonic acid in their structure they have different biosynthetic and degradation routes [49], which suggest they play a different modulatory role. With regard to the modulation of food intake by eCBs, an elegant experiment of Monteleone et. al. [50] showed an increase in 2-AG concentrations but not those of AEA associated to hedonic eating. We also note that although unrelated to differences in olfactory capacity, AEA concentrations were lower in healthy under-weight subjects. This is opposite to the eCB profile in anorexia, as anorexic subjects have elevated concentrations of AEA and unchanged concentrations of 2-AG [51]. Circulating concentrations of eCB-related compounds mimicked the differential trend of eCBs AEA and 2-AG with regard to the BMI of the participants. The collective low concentrations of N-acylethanolamines AEA, LEA, and PEA in under-weight subjects and the collective high concentrations of 2-acylglycerols 2-AG, 2-LG and 2-OG in obese and morbidly obese subjects suggest an up-regulation and/or down-regulation of the enzymes responsible for eCB biosynthesis and inactivation. With the exception of AEA and 2-AG, the rest of N-acylethanolamines and 2-acylglycerols do not bind to CB1 or CB2 receptors and have different molecular targets. However, they are biosynthesized and degraded by the same enzymes that metabolize AEA and 2-AG respectively [52]. In obese subjects, an up-regulation of diacylglycerol lipase α (DAGLα), the enzyme that biosynthesizes 2-AG and the other 2-acylglycerols, and a down-regulation of monoacylglycerol lipase (MAGL), the enzyme that degrades 2-AG and other 2-acylglycerols, have been reported [53]. Studies also have reported that a down-regulation of fatty acid amide hydrolase (FAAH), the enzyme that degrades AEA and other N-acylethanolamines, is associated to obesity [15,53,54]. In our study, however, we did not observe significant differences in N-acylethanolamine concentrations in obese subjects. ECB-related compounds or cannabimimetics may also enhance the activity of AEA and 2-AG by competing with inactivating enzymes or carriers (entourage effect) [55]. Cannabimimetics can bind to different receptors so they can have different or opposite actions to eCBs. These actions are mediated through peripheral receptors. The most studied of the cannabimimetic compounds are OEA, that has anorectic effects, and PEA that has anti-inflammatory effects. Both actions are mediated by activation of peripheral peroxisome proliferator-activated receptor-alpha (PPARα), which is highly expressed in metabolically active tissues [56]. In obesity, the biosynthesis of N-acylethanolamines PEA and OEA may also be dysregulated in peripheral tissues such as adipose tissue and pancreas [57]. PEA is also the cannabimimetic compound more abundant in adipocytes [58]. OEA, PEA, LEA, 2-OG and other 2-acylglycerols are possible endogenous agonists of GPR119, which acts as a fat sensor. The activation of this receptor stimulates the release of intestinal glucagon-like peptide 1 (GLP-1), an incretin involved in glucose homeostasis and satiation [59–61]. 2-acylglycerols originate from fat digestion in the intestinal lumen, from the hydrolysis of triacylglycerol [62]. The high plasma concentrations of 2-acylglycerols 2-LG and 2-OG that we found in obese subjects may be related to the high triglyceride plasma concentrations also found in obese subjects.

In summary we have shown that obese subjects have a lower olfactory capacity than non-obese ones and that high 2-AG fasting plasma concentrations are linked to a lower olfactory capacity. In agreement with previous studies we showed that elevated 2-AG concentrations were linked to high BMI and obesity. Obese subjects had deficits in both odor threshold (OT) and odor identification (OI) capacity. A decrease in OI or in OD capacity may be related to cognitive deficits associated to obesity. Lower AEA and other NAE concentrations were associated to low BMI, while high 2-AG and other 2-MG concentrations were associated to high BMI, suggesting a possible differential up-regulation or down-regulation of the enzymes that biosynthesize and inactivate NAE and 2-MG in these two extreme weight categories.

We cannot yet link a lower olfactory capacity as a result of a dysregulated eCB system in obesity since this is a cross-over study, and a cause-effect cannot be inferred from these data. This study has also other limitations that need to be addressed in future studies. First, the conclusions cannot be applied to the general population, and only to women. These findings should be replicated in men. Second, the relationship between plasma and brain eCB concentrations is unknown. We make the assumption that peripheral eCB concentrations are biomarkers of the state of the brain eCB system. The origin of plasma eCB is unknown and they may be originated in part from organ spillover. Third, eCB concentrations were measured after 12 hours fasting conditions and olfactory tests were performed after at least one hour of fast in the same morning. Despite these time differences we think our results are still valid since they were obtained in separate standardized conditions for all subjects and we were able to find significant differences between groups in both olfactory capacity and eCB concentrations. A simultaneous measure in time of plasma eCBs and olfactory performance is also not possible to obtain since it takes at least half an hour for a subject to undertake the olfactory test. And last, future studies should also include food-related odors due to their known effects on olfactory thresholds, and for the study of brain reward circuits and food enjoyment.

## Supporting Information

S1 Dataset (SAV)
